# Association between the ratio of triglycerides to high-density lipoprotein cholesterol and nocturnal hypertension: a cross-sectional study in a Chinese population

**DOI:** 10.3389/fendo.2025.1474467

**Published:** 2025-05-29

**Authors:** Hezeng Dong, Zhaozheng Liu, Jinling Zhang, Ye Han, Jing Zhang, Yazhi Xi, Liping Chang, Yue Deng

**Affiliations:** ^1^ College of Traditional Chinese Medicine, Changchun University of Traditional Chinese Medicine, Changchun, Jilin, China; ^2^ Heart Disease Center, Hospital Affiliated to Changchun University of Traditional Chinese Medicine, Changchun, Jilin, China

**Keywords:** nocturnal hypertension, TG/HDL-C, ambulatory blood pressure, Asian, cross-sectional studies

## Abstract

**Background and objective:**

The ratio of triglycerides to high-density lipoprotein cholesterol (TG/HDL-C) serves as a predictive indicator for metabolic syndrome and cardiovascular diseases. Simultaneously, nocturnal hypertension significantly increases the risk of target organ damage and cardiovascular events. However, the relationship between the TG/HDL-C ratio and nocturnal hypertension remains unclear. Therefore, the present study aimed to determine the efficacy of the TG/HDL-C ratio in predicting the occurrence of nocturnal hypertension and reducing related adverse events.

**Method:**

Our rigorous cross-sectional study, which included 749 participants who underwent 24-hour ambulatory blood pressure monitoring at the Cardiology Center of Changchun University of Chinese Medicine Affiliated Hospital, allowed us to determine the association between the TG/HDL-C ratio and nocturnal hypertension. We employed both univariate and multivariate logistic regression analyses to ensure the robustness of our findings. Logistic regression modeling was used to assess the independent predictive ability of TG/HDL-C for nocturnal hypertension while adjusting for confounders such as sex, age, BMI, and smoking status. Model performance was assessed by subject work characteristics (ROC) curves and area under the curve (AUC).

**Results:**

Among the 749 participants included in this study, 566 were identified with nocturnal hypertension. Univariate logistic regression analysis demonstrated that the TG/HDL-C ratio was positively correlated with the occurrence of nocturnal hypertension, with the risk of nocturnal hypertension increasing by 24% (OR 1.24(1.06-1.45), P=0.006) for every 1-unit increase in the TG/HDL-C ratio. After adjusting for past medical history, medication, and other relevant examinations, a multivariate analysis revealed a significant correlation between the TG/HDL-C ratio and nocturnal hypertension. Logistic regression analysis demonstrated that TG/HDL-C was positively associated with nocturnal hypertension (regression coefficient = 0.115, P < 0.05). After adjusting for sex, age, BMI, and smoking status, TG/HDL-C remained a predictor of nocturnal hypertension.

**Conclusion:**

Our study underscores the significant association between an elevated TG/HDL-C ratio and the occurrence of nocturnal hypertension. This finding has the potential to draw the attention of patients and physicians to lipid levels, particularly among males, individuals over 45 years old, those with a BMI greater than 24, smokers, and those with a history of hypertension. An independent positive association between TG/HDL-C and nocturnal hypertension was also determined using logistic regression modeling. The findings suggests that it may have potential application in the early screening of nocturnal hypertension. However, the predictive ability is limited, and further studies are necessary to incorporate larger sample sizes and longitudinal designs. These additional studies would validate the predictive role of TG/HDL-C and explore its biological mechanisms.

## Background

In 2023, the World Health Organization released its inaugural *Global Hypertension Report*, which clarified that hypertension affects more than one-third of adults worldwide, with most patients remaining unaware of their condition. If not controlled in a timely manner, hypertension can cause damage to multiple systems throughout the body (1). Recent advancements in hypertension research have led to increased attention to the fluctuations in nocturnal blood pressure. However, large-scale epidemiological studies on nocturnal hypertension remain limited. According to a statement from the American Heart Association, nocturnal hypertension can significantly increase the risk of target organ damage and cardiovascular events (2). A meta-analysis conducted on individual data from 3,468 patients across four prospective European studies revealed that nocturnal blood pressure fluctuations are more predictive of cardiovascular mortality in hypertensive patients than changes in daytime blood pressure (3). Furthermore, an epidemiological study in South Korea indicated that nocturnal hypertension not only affects the diastolic function of the heart in the general population but also increases the risk of white matter hyperintensities (4) in the brain, thereby exacerbating cerebrovascular disease. The incidence rate of nocturnal hypertension in China is considerably higher than that in Europe, which can be attributed to the impact of dietary structure and other factors on diurnal blood pressure (5). Despite the availability of 24-hour ambulatory blood pressure monitoring (ABPM), there is still a lack of predictive indicators for the occurrence of nocturnal hypertension in current clinical practice, particularly in hypertensive patients.

The ratio of TG/HDL-C is a novel clinical indicator commonly used to evaluate insulin resistance due to its relatively simple screening process (6). Recent studies have shown that this indicator is associated with a variety of cardiovascular diseases, which may be attributed to long-term lipid metabolism disorders that lead to and aggravate atherosclerosis (7). However, the correlation between the TG/HDL-C ratio and nocturnal hypertension has been rarely reported. Understanding the prevalence of hypertension among Asian populations and their distinct dietary habits underscores the importance of studying nocturnal hypertension. Moreover, the timely detection of nocturnal hypertension during risk factor screening is essential for adjusting medication appropriately, thereby reducing the risk of nocturnal unconsciousness and cerebrovascular events.

## Materials and methods

**Figure 1:**
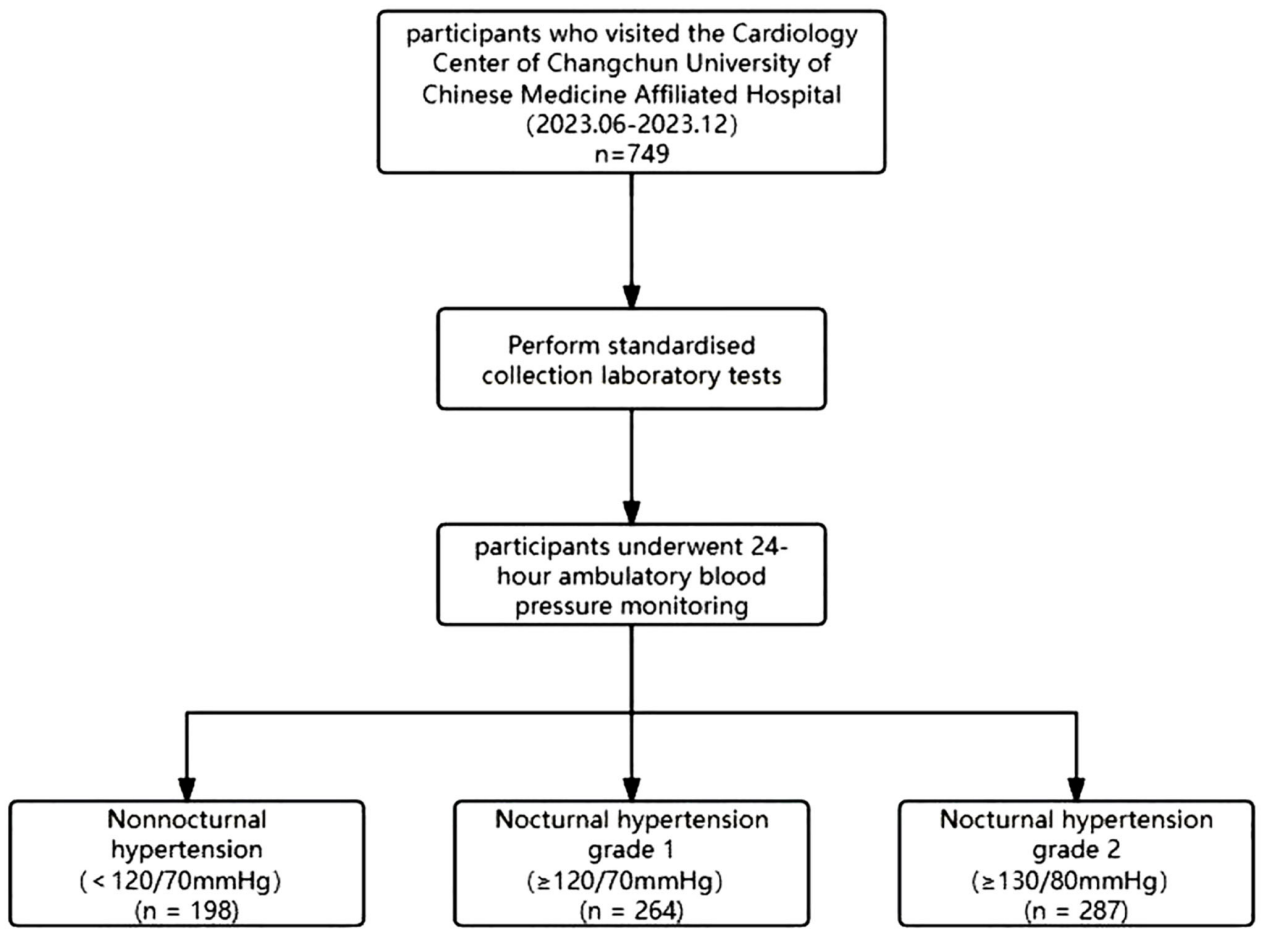
Flowchart for selecting participants.

This study included 749 patients who underwent 24-hour ambulatory blood pressure monitoring at the Cardiology Department of Changchun University of Chinese Medicine Affiliated Hospital between June 2023 and December 2023(**Figure 1**). Exclusion criteria for this study were unclear data or missing and incorrect data, and demographic data, such as sex, age, height, weight, and heart rate, collected upon admission. Patients were defined as smokers if they had smoked continuously or cumulatively for 6 months or more in the past. Patients were defined as alcohol drinkers if they had consumed alcohol ≥1 drink per week in the past 12 months. patients with diabetes if their fasting blood glucose concentration was above 6.1mmol/L, their glycosylated hemoglobin level exceeded 6.5%, or if they were currently using insulin or oral hypoglycemic agents. Hypertension was defined as systolic blood pressure ≥ 140mmHg, diastolic blood pressure ≥ 90mmHg, or use of antihypertensive medication. According to the Chinese Guidelines for Blood Lipid Management (8), patients underwent at least two standard fasting venous blood lipid tests, with a 2-week interval between each test. A diagnosis of abnormal blood lipid levels was made if one or more of the following criteria were met: total serum cholesterol level ≥ 6.2mmol/L, low-density lipoprotein cholesterol ≥ 4.1mmol/L, or triglycerides ≥ 2.3mmol/L. Based on the medications taken within the past 3 months, specific drug information can be determined, including blood pressure medications and statin drugs. The diagnostic thresholds for ambulatory BP were defined according to the ESH hypertension guidelines and the Chinese guidelines for ambulatory BP monitoring (9). The stage 1 threshold for nocturnal BP, corresponding to an office BP of 140/90 mmHg, was 120/70mmHg; the stage 2 threshold for nocturnal hypertension was defined as 130/80 mmHg. This retrospective study was carried out using the opt-out method for the case series of our hospital. To further assess the independent predictive ability of TG/HDL-C for nocturnal hypertension by logistic regression modeling, we identified two datasets that contained the following variables: Outcome Variable: Nocturnal hypertension (0=negative,1= positive).Predictor Variables: TG/HDL-C (ratio of triglycerides to high-density lipoprotein cholesterol), sex, age (years), BMI (kg/m²), and smoking status (0= non-smoker, 1= smoker).Continuous variables (TG/HDL-C, age, and BMI) were standardized using z-scores to ensure consistency across feature scales. The dataset was randomly divided into training (70%) and testing (30%) sets for model development and validation.

The study was approved by the Ethics Committee of the Affiliated Hospital of Changchun University of Chinese Medicine and was conducted in accordance with the 1964 Helsinki Declaration and its later amendments or comparable ethical standards. Informed consent was waived by our Institutional Review Board because of the retrospective nature of our study.

### Measurement of Ambulatory Blood Pressure and Determination of Endpoint

The ambulatory blood pressure recorder used by the Cardiology Center of the Changchun University of Chinese Medicine Affiliated Hospital was sourced from Wuxi Zhongjian Science Instrument Co., Ltd. (model: CB-1805-B, registration number: SXZZ 20142070640). The collected data were analyzed using the ARCS system (Version ABP9.1.1), enabling retrospective analysis of ambulatory blood pressure. We set monitoring intervals from 6:00 to 22:00 during the day, taking blood pressure readings every 30 min. At night, monitoring was continued from 22:00 to 06:00, and measurements were recorded hourly. The reading was considered valid if the number of measurements taken at night was greater than seven. We obtained the average daytime and nocturnal blood pressure values using software analysis. This study was conducted to define nocturnal hypertension according to the ESH Hypertension Guidelines 2023 and Chinese Guidelines for the Prevention and Control of Hypertension. Nocturnal hypertension was defined as an average nighttime systolic blood pressure recorded by ambulatory blood pressure monitoring (ABPM) of ≥120 and/or diastolic blood pressure of ≥70 mmHg, regardless of their daytime blood pressure status, arytenoid or non-arrytenoid (10, 11).

### Laboratory test

We conducted all laboratory tests on fasting participants upon admission, using venous blood collection. The collection process and testing methods were performed under standard conditions, focusing on blood lipid testing, which included total cholesterol, triglycerides, apolipoprotein A1, apolipoprotein B, high-density lipoprotein, low-density lipoprotein, and lipoprotein a. Additionally, we calculated the ratio of apolipoprotein B to apolipoprotein A1 and the ratio of triglycerides to high-density lipoprotein cholesterol based on the results following established standards. We retrospectively collected all the data using a standardized data collection table.

### Statistical analysis

All analyses were performed using the statistical software Free Statistics, version 1.9. Bilateral P values < 0.05 were considered statistically significant. Categorical variables are represented by numbers (n) and percentages (%) and were evaluated using chi-square tests. The mean ± standard deviation of a normal distribution represents continuous variables. We included each variable in univariate and multivariate logistic analyses to evaluate the association between the TG/HDL ratio and nocturnal hypertension. We validated the robustness of the results by adjusting for confounding factors. A logistic regression model was employed to assess the independent predictive ability of TG/HDL-C for nocturnal hypertension while adjusting for confounding variables (sex, age, BMI, and smoking status). The model’s performance was evaluated using the receiver operating characteristic (ROC) curve and the area under the curve (AUC). The AUC reflects the model’s ability to discriminate between positive and negative cases.

## Results

Participants were classified based on the presence of nocturnal hypertension and average nocturnal blood pressure. The absence of nocturnal hypertension (n=198) was defined as nighttime mean systolic blood pressure<120mmHg and/or diastolic blood pressure<70mmHg. Nocturnal hypertension level 1 (n=264) was defined as nocturnal mean systolic blood pressure≥120mmHg and/or diastolic blood pressure≥ 70mmHg. Nocturnal hypertension level 2 (n=287) was defined as nocturnal mean systolic blood pressure≥ 130mmHg and/or diastolic blood pressure≥ 80mmHg. We summarized the characteristics of the participants, represented categorical variables by numbers (n) and percentages (%), and evaluated them using the chi-square test. **Table 1** details the representation of continuous variables as the mean and standard deviation of the normally distributed data.

**Table 1:** Baseline characteristics of all participants.

Variables	All participants (n = 749)	Nonnocturnal hypertension (<120/70mmHg) (n = 198)	Nocturnal hypertension grade 1 (≥120/70mmHg) (n = 264)	Nocturnal hypertension grade 2 (≥130/80mmHg) (n = 287)	p	statistic
Sex, n (%)					**0.026**	7.279
female	450 (60.1)	131 (66.2)	163 (61.7)	156 (54.4)		
male	299 (39.9)	67 (33.8)	101 (38.3)	131 (45.6)		
Age Mean ± SD	64.3 ± 11.5	64.3 ± 11.0	63.6 ± 11.4	64.9 ± 11.9	0.453	0.793
High (m) Mean ± SD	1.6 ± 0.1	1.6 ± 0.1	1.6 ± 0.1	1.7 ± 0.1	0.244	1.411
Weight (kg) Mean ± SD	67.7 ± 12.7	64.3 ± 11.1	67.0 ± 11.5	70.6 ± 14.2	**< 0.001**	15.173
BMI (kg/m2) Mean ± SD	24.8 ± 3.6	23.8 ± 3.5	24.7 ± 3.2	25.7 ± 3.8	**< 0.001**	16.466
Heart.rate (bpm) Mean ± SD	77.9 ± 13.7	76.8 ± 13.6	78.6 ± 13.6	78.1 ± 13.9	0.383	0.961
ACEI/ARB n (%)					**0.001**	13.751
No	604 (80.6)	177 (89.4)	208 (78.8)	219 (76.3)		
Yes	145 (19.4)	21 (10.6)	56 (21.2)	68 (23.7)		
β-blocker n (%)					0.369	1.994
No	580 (77.4)	154 (77.8)	211 (79.9)	215 (74.9)		
Yes	169 (22.6)	44 (22.2)	53 (20.1)	72 (25.1)		
CCB, n (%)					**< 0.001**	29.427
No	484 (64.6)	157 (79.3)	168 (63.6)	159 (55.4)		
Yes	265 (35.4)	41 (20.7)	96 (36.4)	128 (44.6)		
Diuretic n (%)					0.828	0.377
No	704 (94.0)	185 (93.4)	250 (94.7)	269 (93.7)		
Yes	45 (6.0)	13 (6.6)	14 (5.3)	18 (6.3)		
ARNI n (%)					0.332	2.207
No	709 (94.7)	187 (94.4)	254 (96.2)	268 (93.4)		
Yes	40 (5.3)	11 (5.6)	10 (3.8)	19 (6.6)		
Statin n (%)					0.12	4.235
No		155 (78.3)	211 (79.9)	209 (72.8)		
Yes	174 (23.2)	43 (21.7)	53 (20.1)	78 (27.2)		
Diabetes n (%)					**0.006**	10.292
No	607 (81.0)	172 (86.9)	218 (82.6)	217 (75.6)		
Yes	142 (19.0)	26 (13.1)	46 (17.4)	70 (24.4)		
Hypertension n (%)					**< 0.001**	54.722
No	224 (29.9)	97 (49)	76 (28.8)	51 (17.8)		
Yes	525 (70.1)	101 (51)	188 (71.2)	236 (82.2)		
Arrhythmia n (%)					0.147	3.837
No	511 (68.2)	131 (66.2)	192 (72.7)	188 (65.5)		
Yes	238 (31.8)	67 (33.8)	72 (27.3)	99 (34.5)		
CAD n (%)					0.197	3.246
No	352 (47.0)	97 (49)	132 (50)	123 (42.9)		
Yes	397 (53.0)	101 (51)	132 (50)	164 (57.1)		
Dyslipidemia, n (%)					0.129	4.103
No	348 (46.5)	99 (50)	129 (48.9)	120 (41.8)		
Yes	401 (53.5)	99 (50)	135 (51.1)	167 (58.2)		
Smoking n (%)					**0.019**	7.93
No	413 (55.1)	120 (60.6)	153 (58)	140 (48.8)		
Yes	336 (44.9)	78 (39.4)	111 (42)	147 (51.2)		
Drinker, n (%)					**0.007**	9.89
No	507 (67.7)	145 (73.2)	187 (70.8)	175 (61)		
Yes	242 (32.3)	53 (26.8)	77 (29.2)	112 (39)		
TC (mmol/L) Mean ± SD	4.9 ± 1.1	4.9 ± 1.2	4.8 ± 1.1	4.9 ± 1.1	0.522	0.652
TG(mmol/L) Mean ± SD	1.9 ± 1.2	1.7 ± 1.1	1.8 ± 1.2	2.1 ± 1.3	**0.012**	4.471
ApoA1 (g/L) Mean ± SD	1.3 ± 0.2	1.3 ± 0.2	1.3 ± 0.2	1.3 ± 0.2	0.175	1.749
ApoB (g/L) Mean ± SD	0.9 ± 0.2	0.9 ± 0.2	0.9 ± 0.2	0.9 ± 0.2	0.097	2.336
ApoB/ApoA1 (mmol/L) Mean ± SD	0.7 ± 0.2	0.7 ± 0.2	0.7 ± 0.2	0.7 ± 0.2	**0.047**	3.067
HDL-C (mmol/L) Mean ± SD	1.2 ± 0.3	1.2 ± 0.3	1.2 ± 0.3	1.2 ± 0.3	**< 0.001**	7.098
TG/HDL-C (mmol/L) Mean ± SD	1.7 ± 1.4	1.5 ± 1.2	1.7 ± 1.5	1.9 ± 1.4	**0.011**	4.538
LDL-C (mmol/L) Mean ± SD	2.9 ± 0.9	3.0 ± 0.9	2.9 ± 0.9	2.9 ± 0.9	0.344	1.068
LPa (mg/L) Mean ± SD	260.5 ± 240.8	255.2 ± 218.3	273.8 ± 250.8	252.0 ± 246.4	0.533	0.63
Nocturnal hypertension, n (%)					**< 0.001**	660.239
no	183 (24.4)	183 (91)	0 (0)	0 (0)		
yes	566 (75.6)	18 (9)	263 (100)	285 (100)		

Data are shown as mean ± standard deviation (SD) for continuous variables and proportions (%)for categorical variables.

Sex, Age, High, Weight, BMI, ACEI/ARB, β-blocker, CCB, Diuretic, ARNI, Statin, Diabetes, Hypertension, Arrhythmia, CAD=Coronary Artery Disease, Dyslipidemia, Smoking, Drink, TC, total cholesterol; TG, triglyceride; ApoA1, apolipoproteinA1; ApoB, apolipoproteinB; HDL-C, high density lipoprotein cholesterol; LDL-C, low density lipoprotein cholesterin; LPa, Lipoprotein a P values in bold are<0.05.

We conducted a univariate analysis to investigate the relationship between TG/HDL-C ratio and nocturnal hypertension. Factors with a p-value of < 0.05. **Table 1**) were identified based on their clinical relevance and prior literature analysis. The results indicated that weight, BMI, TG, HDL-C, and the TG/HDL-C ratio were significantly correlated with nocturnal hypertension. The TG/HDL-C ratio, the main indicator of this study, was found to increase the risk of nocturnal hypertension by 24% (OR 1.24, P=0.006) for every 1 unit increase. **Table 2** provides the detailed information.

**Table 2:** Univariate analysis for overall population.

Variable	OR_95CI	P_value
Sex=male	1.41 (0.99~1.99)	0.056
Age	1 (0.98~1.01)	0.773
Weight (kg)	1.03 (1.02~1.05)	**<0.001**
BMI (kg/m2)	1.14 (1.08~1.19)	**<0.001**
Heart.rate (bpm)	1.01 (1~1.02)	0.168
Smoking=yes	1.39 (0.99~1.95)	0.058
Drinker=yes	1.37 (0.94~1.97)	0.098
TC (mmol/L)	0.96 (0.83~1.11)	0.606
TG (mmol/L)	1.26 (1.06~1.49)	**0.009**
ApoA1 (mmol/L)	0.75 (0.32~1.78)	0.518
ApoB (mmol/L)	0.87 (0.42~1.82)	0.72
ApoB/ApoA1 (mmol/L)	1.01 (0.44~2.35)	0.981
HDL-C (mmol/L)	0.58 (0.34~0.98)	**0.041**
TG/HDL-C (mmol/L)	1.24 (1.06~1.45)	**0.006**
LDL-C (mmol/L)	0.94 (0.78~1.12)	0.478
LPa (mg/L)	1 (1~1)	0.695

OR, odds ratio; CI, confidence interval; SD, standard deviation.

Abbreviations as in **Table 1**.

P values in bold are < 0.05.

**Figure 2:**
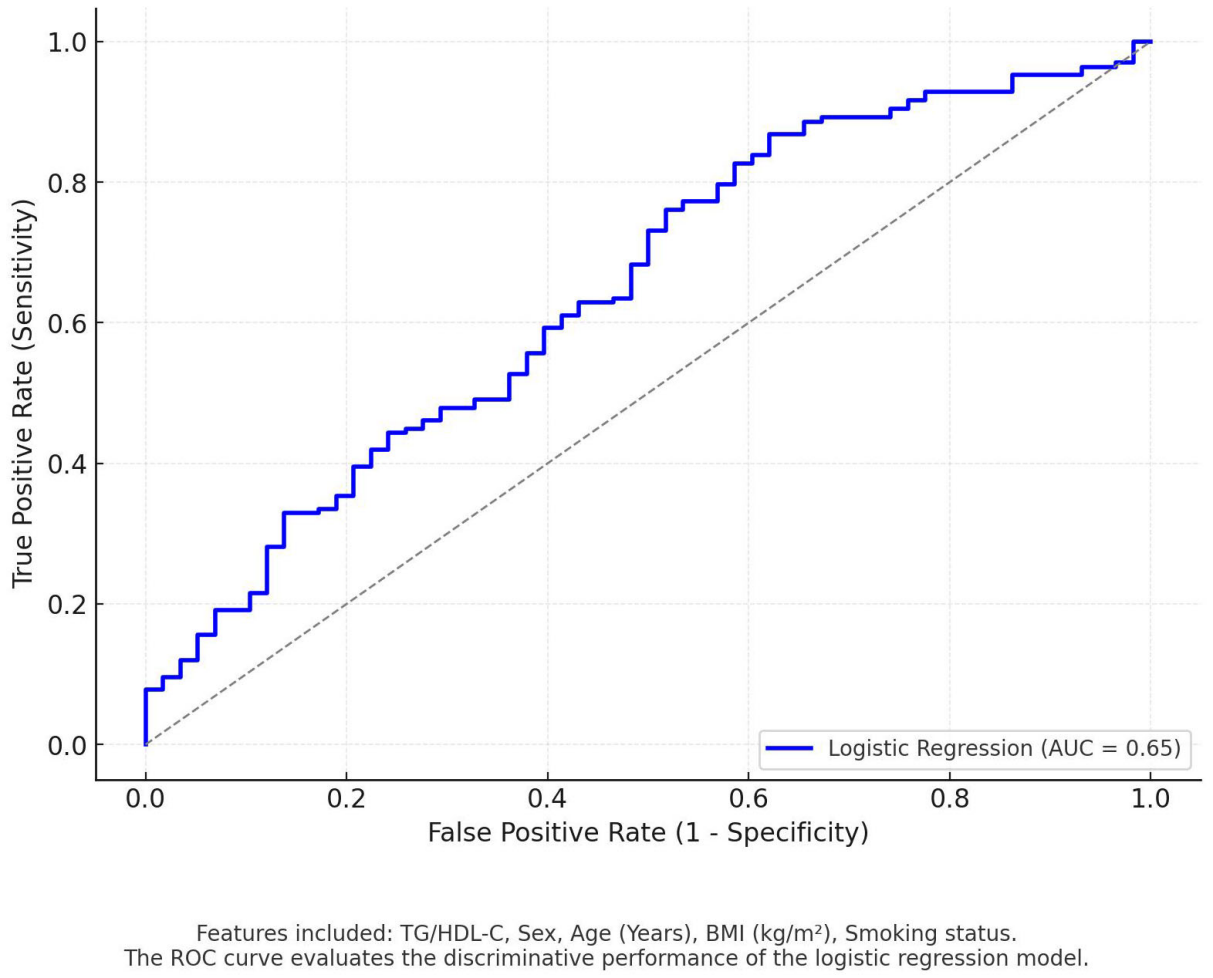
ROC curve: logistic regression predicting nocturnal hypertension.

We performed multivariate analysis to assess the robustness of the results against confounding factors. Model 1 is not adjusted. Model 2 introduced participants’ medical histories, including diabetes, hypertension, arrhythmia, CAD, dyslipidemia, smoking, and alcohol drinkers, and recognized their impact on outcomes. Model 3 was further adjusted for past medication information, such as ACEI/ARB, β-blocker, CCB, diuretic, ARNI, and statin, which could affect the changes in blood pressure and lipid levels. The analysis demonstrated that the relationship between TG/HDL-C and nocturnal hypertension remained robust in various models considering past history, medication factors, and other physicochemical indicators (**Table 3**), further verifying the research results. The logistic regression model achieved an AUC of 0.65 on the test set (**Figure 2**).

**Table 3:** multivariate analysis for overall population.

Variable	Model 1	Model 2	Model 3
n.total	749	749	749
n.event_%	566 (75.6)	566 (75.6)	566 (75.6)
crude.OR_95CI	1.24 (1.06~1.45)	1.24 (1.06~1.45)	1.24 (1.06~1.45)
crude.P_value	0.006	0.006	0.006
adj.OR_95CI	1.14 (1.02~1.35)	1.18 (1.01~1.38)	1.2 (1.02~1.4)
adj.P_value	0.043	0.036	0.025

Model1-Adj: Basic Information (years, sex, BMI, somking, drink).

Model2-Adj: Past medical history (diabetes, hypertension, arrhythmia, CAD, dyslipidemia).

Model3-Adj: Medications (ACEI/ARB, β-blocker, CCB, diuretic, ARNI, statin).

### Subgroup analysis

We conducted a subgroup analysis to further investigate the relationship between the TG/HDL-C ratio and nocturnal hypertension across different patient subgroups. We stratified participants based on age (≥45 and <45 years), gender, obesity status (BMI ≥24 kg/m^2^ and <24 kg/m^2^, according to the Chinese BMI standard) (12), smoking history, and history of hypertension. The analysis revealed a more pronounced association between the TG/HDL-C ratio and nocturnal hypertension among individuals aged 45 or older, males, those with a BMI ≥24 kg/m2, smokers, and those with a history of hypertension (**Table 4**).

**Table 4:** Subgroup analysis for association between TG/HDL-C and nocturnal hypertension.

Subgroup	n. Total	n. event%	crude. OR_95CI	crude.Pvalue	P.for.interaction1	P.for.interaction2	adj. OR_95CI	adj.P value	P.for.inte raction1	P.for.inter action_2
Sex										
sex=female	450	329 (73.1)	1.14 (0.95~1.36)	0.174	0.169	0.182	1.1 (0.91~1.33)	0.306	0.14	0.154
sex=male	299	237 (79.3)	1.43 (1.07~1.91)	**0.014**			1.35 (1.01~1.82)	**0.044**		
Age										
Age<45	40	29 (72.5)	1.51 (0.75~3.04)	0.243	0.554	0.571	1.63 (0.71~3.74)	0.246	0.486	0.505
Age≥45	709	537 (75.7)	1.23 (1.05~1.44)	**0.01**			1.18 (1.01~1.39)	**0.039**		
BMI										
BMI<24kg/m2	298	202 (67.8)	1.05 (0.84~1.32)	0.676	0.205	0.207	0.98 (0.78~1.24)	0.882	0.225	0.228
BMI≥24kg/m2	451	364 (80.7)	1.29 (1.03~1.6)	**0.025**			1.26 (1.01~1.57)	**0.041**		
Hypertension										
hypertension= no	224	133 (59.4)	1.1 (0.89~1.36)	0.395	0.265	0.267	1.1 (0.89~1.37)	0.381	0.318	0.321
hypertension= yes	525	433 (82.5)	1.31 (1.04~1.64)	**0.02**			1.29 (1.02~1.63)	**0.032**		
Smoking										
smoke=no	413	301 (72.9)	1.16 (0.96~1.41)	0.129	0.407	0.411	1.13 (0.92~1.37)	0.238	0.378	0.384
smoke=yes	336	265 (78.9)	1.33 (1.03~1.71)	**0.027**			1.29 (1~1.67)	**0.048**		

Covariates included: medication use, height, weight, history of diabetes.

OR, odds ratio; CI, confidence interval; SD, standard deviation. P values in bold are < 0.05.

## Discussion

### Nocturnal hypertension: high prevalence and dangers

Nocturnal hypertension has garnered increased attention from researchers in recent years. However, the prevalence of this condition across different regions and ethnic groups remains unclear due to the lack of large-scale epidemiological studies. In the present study, more than half of the participants exhibited elevated nighttime blood pressure, which is consistent with the findings of de la Sierra et al. in their study, which included 37,096 untreated patients from the Spanish Ambulatory Blood Pressure Monitoring Registry and 62,788 patients receiving anti hypertensive treatment, 40.9% of the untreated group and 49.8% of the treatment group experienced nocturnal hypertension (13). Similarly, JM et al. and Ma et al. conducted ambulatory blood pressure monitoring on hypertensive patients and discovered that more than half of the study population had significant nocturnal hypertension (14, 15).Nocturnal hypertension poses a greater threat compared to daytime fluctuations, primarily due to the difficulty of timely detection and treatment. A substantial body of prior research has centered on identifying predictors of elevated daytime blood pressure. Our study builds upon this research by extending the scope to nocturnal hypertension, a condition that has been demonstrated to exhibit a more robust correlation with target organ damage and cardiovascular events. Elevated nighttime blood pressure demonstrates a stronger correlation with systemic diseases such as cognitive impairment and cerebrovascular conditions. Kario et al. proposed that patients with elevated nighttime blood pressure have a poorer prognosis for both stroke and cardiovascular events compared to those with elevated daytime blood pressure (16). These findings indicate that elevated nighttime blood pressure can cause damage across multiple bodily systems (3, 17). Hoshide et al.’s study also suggested that even in hypertensive patients with well-controlled self-measured home blood pressure, dynamic nocturnal blood pressure elevation may promote target organ damage (18).

### Relationship between TG/HDL-C ratio and diseases

This study explored the relationship between the ratio of triglycerides to high-density lipoprotein cholesterol (TG/HDL-C) and nocturnal hypertension in a Chinese population. Our findings indicate that the TG/HDL-C ratio is positively associated with the risk of nocturnal hypertension. This suggests that TG/HDL-C, a marker of metabolic dysregulation, may serve as a useful indicator for identifying individuals at risk for nocturnal hypertension, especially in populations with a high prevalence of metabolic disorders. In recent medical studies, an increasing number of researchers have identified metabolic markers as predictors of elevated blood pressure, and a large cohort study in China found that SUA levels were an independent predictor of blood pressure progression and the development of hypertension in the Chinese population (19). A cross-sectional study of an HIV program in Argentina found that INH was highly prevalent in PWH. Metabolic and inflammatory markers predict nocturnal SBP in PWH (20). The ratio of triglyceride (TG) to high-density lipoprotein cholesterol (HDL-C) has consistently been recognized as a significant indicator of metabolic syndrome. Extensive research has demonstrated that the TG/HDL-C ratio surpasses individual lipid markers, such as low-density lipoprotein cholesterol (LDL-C), HDL-C, or TG, in predicting the development of type 2 diabetes over a 10-year period (21).The present findings are consistent with the results of other studies that have emphasized the role of TG/HDL-C ratio as a marker of cardiovascular risk, and elevated TG/HDL-C ratio has been associated with insulin resistance, dyslipidemia, and arterial stiffness (22), all of which are associated with hypertension. Furthermore, the TG/HDL-C ratio has been identified as a superior indicator of metabolic syndrome in obese children and adolescents (23). In recent years, a growing body of evidence has highlighted TG/HDL-C ratio as a crucial predictor of various cardiovascular diseases. Azarpazhooh MR et al. reached a similar conclusion, stating that the TG/HDL-C ratio can effectively identify patients with metabolic syndrome, insulin resistance, and severe atherosclerosis (24). These findings underscore the significance of the TG/HDL-C ratio as a vital indicator in contemporary clinical diagnosis and expand upon previous research by innovatively proposing the TG/HDL-C ratio as a predictive factor for nocturnal hypertension, addressing the gap in nighttime blood pressure prediction. Logistic regression analysis demonstrated a positive correlation between TG/HDL-C ratio and nocturnal hypertension (regression coefficient of 0.115), indicating that elevated TG/HDL-C ratio is associated with an increased risk of nocturnal hypertension. This observation supports the hypothesis that TG/HDL-C ratio may serve as a potential marker for cardiovascular risk. Despite the relatively modest strength of the observed association, its independent predictive effect remained consistent after controlling for confounding variables, including sex, age, BMI, and smoking status. These findings offer significant insights into the management of hypertension. Daily fluctuations in lipids must be considered in the management of blood pressure in hypertensive patients, leading to timely adjustment of medication strategies. This may necessitate the incorporation of lipid-regulating medications when abnormalities are identified during treatment and the adjustment of medication timing to mitigate the risk of nocturnal hypertension. This integrated approach is expected to address the critical problem of difficulty in detecting and poor control of nocturnal blood pressure elevations in hypertension treatment.

### Specificity of the study population

A prior study focusing on hypertension in Asian populations emphasized the importance of addressing drug resistance and nocturnal hypertension in Asia ^[26]^. The participants included in the present study are all Asian. Considering the aging population and unique dietary habits in Asia, the incidence of hypertension is increasing annually. Therefore, the investigation of nocturnal hypertension in Asia provides significant guidance for the prevention, control, management, and treatment of hypertension in this region.

### Deficiencies and prospects

Given the cross-sectional design of the study and the lack of long-term follow-up, the findings serve as preliminary evidence based on current research, necessitating further investigation for a comprehensive understanding. Furthermore, the modest sample size, in conjunction with the possibility that other variables were not completely incorporated into the model, yielded an AUC value of 0.65 for the resulting logistic regression model. This finding indicates a modest degree of predictive capability. Consequently, it is imperative that we undertake a large-scale study to substantiate the evidence and enhance the robustness of the model. Furthermore, the study population primarily consists of individuals from northern China, with limited representation from other regions. To address these limitations, future research should aim to conduct multicenter, multiethnic systematic studies to enhance the generalizability of the findings and better inform clinical prevention and treatment strategies.

## Conclusion

In conclusion, the present study has definitively established an association between the TG/HDL-C ratio and nocturnal hypertension, even after adjusting for various confounding variables. This association is particularly pronounced among middle-aged and elderly individuals, males, those who are obese, smokers, and individuals with a history of hypertension. The positive correlation between TG/HDL-C and nocturnal hypertension was confirmed by logistic regression modeling. Despite the limitations of the logistic regression model, the findings provide a foundation for further investigation into the predictive mechanisms of nocturnal hypertension. Subsequent studies should integrate additional clinical and lifestyle characteristics and employ more advanced models to enhance predictive capabilities. Considering the significant risk posed by nocturnal hypertension, the results of blood lipid tests should be emphasized in future clinical practice to screen patients with elevated nighttime blood pressure, guide patients to adhere to medication regimens, and prevent adverse nocturnal events.

## Data availability

Data in support of this study were obtained from self-constructed databases and can be obtained from corresponding authors.

## Data Availability

The raw data supporting the conclusions of this article will be made available by the authors, without undue reservation.
